# Minimum dietary diversity and associated factors among lactating mothers in Haryana, India: a community based cross-sectional study

**DOI:** 10.1186/s12887-022-03588-5

**Published:** 2022-09-03

**Authors:** Shumayla Shumayla, E. M. Irfan, Nishtha Kathuria, Suresh Kumar Rathi, Shobhit Srivastava, Sunil Mehra

**Affiliations:** Mamta-Health Institute for Mother and Child, B-5, Greater Kailash-II, Delhi, 110048 India

**Keywords:** Dietary diversity, Lactating mothers, Nutritional knowledge, Haryana

## Abstract

**Introduction:**

Food adequacy and dietary quality in the lactation period are fundamental for maternal and child health. Lactating mothers are vulnerable to malnutrition because of increased physiological demand, monotonous diet, lactogenesis process, and increased nutrient requirements. The micronutrient adequacy especially among women is not ensured in Indian diet. The dual course of gender bias and poverty, along with lack of knowledge about diet quality are significant impediments in maintaining minimum dietary diversity among Indian women. The study aimed to assess the prevalence of minimum dietary diversity and associated factors among lactating women.

**Methodology:**

A community-based cross-sectional study was conducted among 1236 lactating women through a multistage sampling procedure in Haryana state, India. Data were collected in Computer-assisted personal interviewing (CAPI) using a pretested structured interview schedule. Minimum Dietary Diversity for Women by Food and Agriculture Organization (FAO) was used to calculate the minimum dietary diversity.

**Results:**

The mean dietary diversity score among lactating women from the ten food groups was 6.35 ± 2.57 and the prevalence of minimum dietary diversity was 77.1%. The complete model revealed that both individual and household factors can explain the variation in dietary diversity intake. Furthermore, the result of model 2 explained that women aged 31 to 35 years (AOR 5.92,95% (1.87–18.77), graduation and above qualified women (AOR 1.98, 95% (0.96–4.09) and lactating women with high knowledge on nutrition (AOR 2.00, 95% (1.34–4.57) were the significant factors promoting minimum dietary diversity.

**Conclusion:**

Three-fourths of the lactating women reached adequate minimum dietary diversity. Younger age, low educational level, and poor nutritional knowledge were significant constraints to achieving minimum dietary diversity. Further improvement in the minimum dietary diversity among lactating women is very much required. It is also advised that exiting platforms dispersing awareness on nutrition should be supported and strengthened.

## Introduction

Dietary diversity (DD) is the variety of foods consumed over a specific time period that will ensure an adequate intake of nutrients that can support good health and the physical and mental development of individuals [1]. Furthermore, it is widely acknowledged as a critical part of healthy diets [2]. Approximately half of the global population is affected by maternal and child undernutrition and micronutrient deficiencies [3]. Especially in South Asia, with higher nutritional requirements, a vast proportion of lactating mothers are vulnerable to nutritional deficiencies [4–6]. The nutritional needs during lactation period are higher. Indian Council of Medical Research (ICMR) also recommended lactating mothers to have at least 600 extra calories/day [7]. When these requirements are not met, lactating mothers may suffer from malnutrition, in particular micronutrient deficiencies. Besides, a poor diet may influence the quality of the nutrient in breast milk [8, 9]. Additionally, clinical adversities such as anaemia [1] is linked to minimum dietary diversity (MDD) among women of reproductive age [10]. Improving the intake of different food items, especially in the 1st 1000 critical days, is one of the best nutritional interventions to prevent these deficiencies and correct the nutritional vulnerability in women of reproductive age [11]. Good dietary diversity of a lactating women has been associated with the good health, growth and development of their children. A few studies found a substantial correlation between what a lactating women ate and what their children, adolescents and adults ate [12, 13].

Food adequacy and dietary quality in the lactation period are fundamental for maternal and child health [14]. Most South Asian women and women in low resource settings predominantly eat starchy staples with restricted or no animal products, vegetables, and fresh fruits [15, 16]. Lactating mothers are vulnerable to malnutrition because of increased physiological demands, eating an undiversified monotonous diet, and lactogenesis process, particularly in low- and middle-income countries [11]. Besides, the socioeconomic status of the household also affects the dietary pattern (high intakes of salt, saturated fats, and sugar and low intakes of fruit, vegetables, and whole grains), dietary habits, and sufficient intake of nutrients of lactating mothers, which directly influence the diversity of diet. This will also vary among lactating mothers in urban and rural areas, as women in urban poverty affected areas have a lower intake of a variety of fruits and vegetables compared to rural areas because of availability [17]. Additionally, a study conducted in one of the South Asian countries stated that lower educational levels and larger family sizes may likely be associated with the low mean dietary diversity among women [3].

By 2030, Sustainable Development Goal 2 plans to eradicate hunger and all types of malnutrition [18]. Ensuring a diverse and adequate diet could act as the foundation of long-term and sustainable strategies for overcoming global malnutrition [19]. Minimum dietary diversity is one of the most significant reasons for micronutrient deficiencies, also prompting macronutrient shortages [20].

Minimum dietary diversity may range from 55.2 to 87.8%, according to studies conducted in some Asian countries [14, 16]. Dietary diversity as an indirect measure of diet quality reflects the three pillars of food security - availability, utilization, and accessibility [17] and also acts as a “proxy indicator” for micronutrient adequacy of an individual’s diet [3].

Although in India the shift from food security to nutrition security has been well taken through dietary diversification, but the micronutrient adequacy especially among women is not ensured in Indian diet. Gender bias and poverty, as well as a lack of awareness about diet quality, are the most significant impediments to achieving a minimum of dietary diversity among women [19]. According to a study conducted among women in India, dietary diversity increases with higher income, diets, and household awareness [21]. Despite countless research on the prevalence of minimum dietary diversity among pregnant women and at the household level undertaken around the world. There is a scarcity of data on the kind and relative magnitude of minimum dietary diversity among lactating mothers in India. This study aimed to assess the prevalence of minimum dietary diversity and associated factors among lactating women in three districts of Haryana state.

## Material and methods study setting, design and sampling

The study was conducted in three districts of Indian state Haryana which is one of the most national food grain productive state of India [20]. The data were collected in March 2021 from three blocks (A block is a district subdivision, for the purposes of the Rural development department and Panchayati Raj institutes [22]. from each selected district namely Palwal (Block: Hassanpur, Hathin and Hodal), Panipat (Block: Madlauda, Israna and Bapoli) and Jhajjar (Block: Badli, Jhajjar and Salhawas).

The study followed the community-based cross-sectional design and adopted the multistage sampling procedure, with selection of districts as the first stage, followed by the selection of blocks, villages, and households. The villages for data collection were chosen by Probability Proportional to Size (PPS) technique [23], however the participants were randomly selected. The sample size was estimated using the Cochran method, N = Z ^2^ P (1-P)/E ^2^ where Z = 1.96 (95% confidence interval), P = estimated proportion of Lactating women (4875), and E (level of precision) = 0.05. The estimated sample size for the study was 423 in each selected district with a nonresponse rate of 10%. Hence, the total sample size for the study was 1269 but 1238 were considered for analysis.

### Study population

All lactating women aged (18–35) who lived in the three districts namely Palwal, Panipat, Jhajjar for at least 6 months were eligible for the study.

### Operational definitions

“Minimum dietary diversity (MDD)” for reproductive age women by Food and Agriculture Organization (FAO), is defined as the consumption of at least five of the ten food groups specified in the list through the previous day and night (24 hours period) preceding the day of the survey [24].

### Data collection

A household-level survey was conducted among lactating women aged 18–35 years residing in the selected villages and having a child of 0–2 years. A pretested structured interview schedule was used to collect information on the basic socio-demography, knowledge and practices on nutrition and maternal health components and service utilization. All tools were translated in local language, i.e., Hindi. Two-day training was conducted with the data collectors on handling the application, interview technique, ethical considerations, and participant rights; reading and understanding the questions and techniques to reduce under/over reporting while keeping anonymity. Timely monitoring was conducted for maintaining the quality of the data. Supervisors were assigned to randomly back-check and spot-check 10% of all forms.

#### Dependent variable

##### Minimum dietary diversity for women

The minimum dietary diversity for women was utilized to assess the overall dietary quality of respondents as it has been shown to demonstrate adequate nutrient intake [25] and can be utilized as a proxy indicator for estimating nutrient adequacy among lactating females [26]. The Minimum Dietary Diversity for Women (MDD-W) indicator were established on a 10-food group women dietary diversity score. These groups are: Group 1: Grains, white roots and tubers, and plantains Group 2: Pulses (beans, peas and lentils) Group 3: Nuts and seeds Group 4: Milk and milk products Group 5: Meat, poultry and fish Group 6: Eggs Group 7: Dark green leafy vegetable Group 8: Other vitamin A-rich fruits and vegetable Group 9: Other vegetable Group 10: Other fruits. Details of the methods of data collection and scoring guide are reported in [24].

A Dietary diversity score was used to characterize the average dietary intake of lactating women in the study area, which is based on a 24-h dietary recall method. The women were asked to recall all foods consumed on the previous day from the above food groups. ‘Yes’ or ‘No’ responses were noted. A ‘yes’ response received a ‘1’ score, and a ‘no’ response received a ‘0’ score. The women’s dietary diversity score was calculated by adding the scores together, which was further classified into adequate and inadequate dietary diversity based on the MDD-W. Women’s with a diversity score of less than 5 were labelled as having inadequate dietary diversity, while those with scores of 5–10 were labelled as having adequate dietary diversity [24].

#### Independent variable

##### Nutritional knowledge score

The nutrition knowledge score was constructed using 6 variables, which are knowledge on tri-color food, heard of anaemia, heard of Iron Folic Acid supplementation, heard of Albendazole, and causes and preventive measures of anaemia. The first four variables mentioned were dichotomous variables which were recorded as “1” if the response to the question was “Yes” and else “0”. The causes of anaemia variables were recoded as “2” if the respondents knew more than 2 causes, “1” if they knew 1 to 2 causes and “0” if they didn’t know any causes. Similarly, the “preventive measures of anaemia” were recoded as “2” if they knew more than 1 preventive measure, “1” if they knew only one preventive measure and “0” if they didn’t know any. Cumulative score was generated by summing up the scores and categorized into 4 groups. Those who scored 0, 1, 2 to 5, and 6 to 8 fall under ‘No knowledge’, ‘Low knowledge’, ‘Medium knowledge’ and ‘High knowledge’ category, respectively.

### Ethical consideration

Each study participant was provided a written description of the study title, purpose, protocol, duration, possible risks, and benefits. Before the data collection informed written consent was obtained. Ethical clearance was obtained from MAMTA- Health Institute for Mother and Child Ethical Review Board vide Reference No. MERB/March-2021/003.

### Data processing and analysis

The data were collected, cleaned, and double checked for missing values and outliers. Thereafter cleaned data analyzed using SPSS version 26. Univariate analysis was done to determine the socio-demographic profile of the respondents and Pearson chi-square test was used to understand the association of dependent and independent variables. To identify the effect of various independent factors on minimum dietary diversity intake, a binary logistic regression model was applied to estimate the adjusted odds ratio (AOR) and 95% confidence interval (CI) for the association with dependent variable. Independent variables such as age of the respondents, education of the respondent and husband, occupation of the respondent, parity, number of ANC visits, and nutrition knowledge were included in this study. The significance level was set to be at 5% (*p* < 0.05).

## Results

More than half of the respondents were in the age group of 21–25 years (Table 1). Seventy per cent of the lactating women belonged to the lower socioeconomic groups, with 67.4% living in a joint/ extended family. Only 19.2% study participants had a college graduation or above. Only a few (1.9%) of the respondent’s husbands didn’t have proper schooling, while nearly a third held a graduation degree and above. The majority (95.5%) of the respondents were homemakers. More than half of the women were multiparous (52.1%) did not receive four minimum ANC visits. The mean dietary diversity score for the study population from the ten food groups was 6.35 ± 2.57. Furthermore, the percentage of respondents who had adequate dietary diversity, i.e., more than 5 food items, was 77.1%, whereas approximately 12.9% of the respondents had no knowledge on Nutrition (Table 1).

**Table 1:** Percentage distribution of knowledge score indicators (*n* = 1238)

	N	%
Knows what is ‘Tri-color food’ (*N* = 1238)	616	49.8
Heard of Anaemia (*N* = 1238)	571	46.1
Causes of Anaemia (*N* = 571)		
Don’t know any causes	19	3.3
Knows 1 or 2 causes only	497	87.0
Knows more than 2 causes	55	9.7
Preventive measures of Anaemia (*N* = 571)		
Don’t know any preventive measures	23	4.0
Knows one preventive measure only	302	52.9
Knows more than one preventive measures	246	43.1
Heard of Iron folic Acid (*N* = 1238)	902	72.9
Heard of Albendazole (*N* = 1238)	429	34.7

‘Nutrition knowledge scores’ indicator (Table 2) showed that around half of the respondents were aware of the ‘Tricolor food’ and ‘anaemia’. The vast majority (87%) of the respondents knew at least one reason of anaemia. A similar situation existed when it came to anaemia prevention, where 52.9% of respondents were aware of at least one preventive measure. Conversely, nearly three-quarters of the respondents had heard of iron folic acid (IFA), whereas only one-third had heard of Albendazole a deworming tablet (Table 2).

**Table 2:** Background characteristics of the lactating women (*n* = 1238)

Variables	Sample	Percentage
**Age of the respondents (in years)**		
Less than or equal to 20	91	7.4
21 to 25 years	709	57.3
26 to 30 years	377	30.5
31 to 35 years	61	4.9
**Education of the respondent**		
No schooling	115	9.3
Primary	111	9
Upper primary	145	11.7
Secondary	237	19.1
Higher secondary	392	31.7
Graduation or above	238	19.2
**Caste**		
General	658	53.15
SC/ST	362	29.24
OBC	218	17.61
**Religion**		
Hindu	1152	93.1
Other	86	6.9
**Education of the Husband**		
No schooling	24	1.9
Primary	59	4.8
Upper primary	156	12.6
Secondary	228	18.4
Higher secondary	382	30.9
Graduation or above	389	31.4
**Occupation of the respondent**		
Unemployed/ Homemaker	1195	96.5
Employed	43	3.5
**Occupation of the husband**		
Unemployed	224	18.1
Employed	1014	81.9
**Type of Family**		
Joint	835	67.45
Nuclear	403	32.55
**Parity of the respondent**		
Primipara	593	47.9
Multiparous	645	52.1
**Number of ANC Visits**		
Less than 4 ANC Visits	818	66.1
4 or more ANC Visits	420	33.9
**Nutrition knowledge**		
No Knowledge	160	12.9
Low Knowledge	359	29
Medium knowledge	376	30.4
High Knowledge	343	27.7
**Minimum Dietary diversity status**		
Inadequate	83	22.9
Adequate	955	77.1

*ANC* Antenatal care

**Fig. 1:**
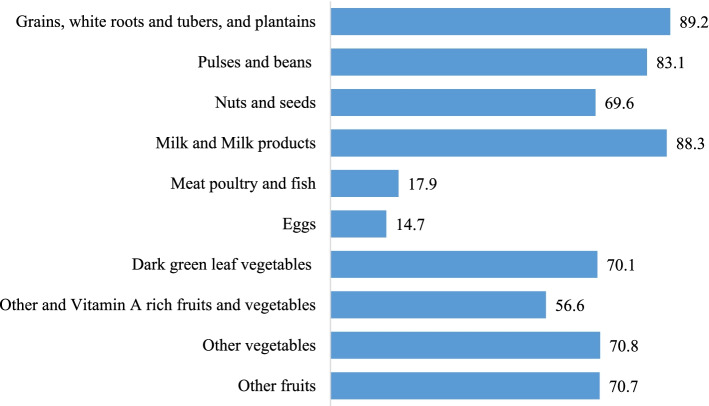
Food intake by the lactating women (*n* = 1238)

Figure 1 showed that starchy staple food was the most commonly consumed food by respondents (89.2%), followed by Milk and milk products (88.3%); while eggs were the least frequently (14.7%) consumed food items.

The percentage of lactating women who had adequate dietary intake was significantly higher among the age group of 31–35 years than their counterparts in other age groups (Table 3). Similarly, education of the respondents, and nutrition knowledge had a significant relation with minimum dietary diversity intake. The percentage of those who had adequate dietary intake was comparatively high among those who pursued graduation and above. Maximum (85%) of lactating women with high nutrition knowledge had adequate dietary diversity (Table 3).

**Table 3:** Background characteristics and dietary diversity among lactating women (*n* = 1238)

Variables	Inadequate n (%)	Adequate n (%)	Chi-square
**Age of the respondents (in years)**			
Less than or equal to 20	30 (33.0)	61 (67.0)	14.73** (0.002)
21 to 25 years	163 (23.0)	546 (77.0)	
26 to 30 years	86 (22.8)	291 (77.2)	
31 to 35 years	4 (6.6)	57 (93.4)	
**Education of the respondent**			
No schooling	29 (25.2)	86 (74.8)	12.85* (0.025)
Primary	28 (25.2)	83 (74.8)	
Upper primary	39 (26.9)	106 (73.1)	
Secondary	68 (28.7)	169 (71.3)	
Higher secondary	78 (19.9)	314 (80.1)	
Graduation or above	41 (17.2)	197 (82.8)	
**Education of the Husband**			
No schooling	7 (29.2)	17 (70.8)	2.106 (0.834)
Primary	12 (20.3)	47 (79.7)	
Upper primary	39 (25.0)	117 (75.0)	
Secondary	54 (23.7)	174 (76.3)	
Higher secondary	80 (20.9)	302 (79.1)	
Graduation or above	91 (23.4)	298 (76.6)	
**Occupation of the respondent**			
Unemployed/ Homemaker	274 (22.9)	921 (77.1)	0.94 (0.759)
Employed	9 (20.9)	34 (79.1)	
**Occupation of the husband**			
Unemployed	52 (23.2)	172 (76.8)	0.02 (0.889)
Employed	231 (22.8)	783 (77.2)	
**Parity**			
Primipara	138 (23.3)	455 (76.7)	0.11 (0.741)
Multiparous	145 (22.5)	500 (77.5)	
**Number of ANC Visits**			
Less than 4 ANC Visits	188 (23.0)	630 (77.0)	0.021 (0.885)
4 or more ANC Visits	95 (22.6)	325 (77.4)	
**Nutrition knowledge**			
No Knowledge	41 (25.6)	119 (74.4)	35.12*** (0.0001)
Low Knowledge	117 (32.6)	242 (67.4)	
Medium knowledge	75 (19.9)	301 (80.1)	
High Knowledge	50 (14.6)	293 (85.4)	

*if *p* < 0.10; **if *p* < 0.05 and ***if *p* < 0.01; *ANC* Antenatal care

Two models of logistic regression were used to understand the best suited factors associated with dietary diversity in Table 4. Individual factors, such as demographic data, are represented in Model 1. Model 1 reflects household characteristics such as cast, family type, and neighborhood, whereas Model 2 represents the overall effect model, which includes both individual and household components. Among the three models, Model 2 has the high R square value, which describes that individual factors along with household factors can explain the variation in dietary diversity intake.

**Table 4:** Logistic regression for dietary diversity intake by associated factors (*n* = 1238)

Variables	Model 1	Model 2
	Adjusted Odds Ratio (95% CI)	Adjusted Odds Ratio (95% CI)
**Age of the respondents (in years)**		
Less than or equal to 20	Reference	Reference
21 to 25 years	1.44 (0.88–2.38)	1.5 (0.91–2.49)
26 to 30 years	1.33 (0.77–2.30)	1.43 (0.81–2.49)
31 to 35 years	5.34** (1.70–16.76)	5.92** (1.87–18.77)
**Education of the respondent**		
No schooling	Reference	Reference
Primary	0.89 (0.47–1.68)	0.96 (0.51–1.83)
Upper primary	0.92 (0.50–1.69)	0.96 (0.52–1.77)
Secondary	0.87 (0.47–1.60)	0.89 (0.48–1.65)
Higher secondary	1.40 (0.74–2.65)	1.45 (0.76–2.76)
Graduation or above	2.04* (1.00–4.13)	1.98* (0.96–4.09)
**Education of the Husband**		
No schooling	Reference	Reference
Primary	2.08 (0.66–6.57)	2.27 (0.71–7.27)
Upper primary	1.61 (0.57–4.49)	1.76 (0.62–4.96)
Secondary	1.45 (0.52–4.06)	1.48 (0.52–4.20)
Higher secondary	1.34 (0.48–3.76)	1.41 (0.49–4.04)
Graduation or above	0.82 (0.28–2.37)	0.84 (0.29–2.46)
**Occupation of the respondent**		
Unemployed/ Homemaker	Reference	Reference
Employed	0.90 (0.41–1.96)	0.92 (0.42–2.01)
**Occupation of the husband**		
Unemployed	Reference	Reference
Employed	0.98 (0.68–1.41)	1.02 (0.69–1.51)
**Parity**		
Primipara	Reference	Reference
Multiparous	1.23 (0.91–1.66)	1.20 (0.89–1.63)
**Number of ANC Visits**		
Less than 4 ANC Visits	Reference	Reference
4 or more ANC Visits	0.87 (0.64–1.17)	0.91 (0.67–1.23)
**Nutrition knowledge**		
No Knowledge	Reference	Reference
Low Knowledge	0.70 (0.45–1.07)	0.72 (0.47–1.12)
Medium knowledge	1.36 (0.85–2.16)	1.59 (0.96–2.63)
High Knowledge	2.00** (1.19–3.38)	2.48** (1.34–4.57)

Model 1 and 2 were adjusted for household characteristics (caste, family type and district); *if *p* < 0.10; **if *p* < 0.05 and ***if *p* < 0.01; *ANC* Antenatal care

After adjusting for the possible confounding variables, higher age of the respondent, the formal education of the respondent, and high nutrition knowledge remained significant and independent predictors of minimum dietary diversity score. Lactating women aged 31 to 35 years were 5.9 times significantly more likely of having adequate dietary diversity than those who were in the age group of less than or equal to 20 years [AOR: 5.92, 95%; CI: 1.87–18.77]. Moreover, women who had graduation and above qualification are twice likely to have adequate dietary diversity intake compared to women with no schooling [AOR: 2.04, 95%; CI: 1.00–4.13]. Lactating women with high knowledge on nutrition had 2.48 times higher odds [AOR: 2.48, 95%; CI: 1.34–4.57] to have adequate dietary diversity intake than the respondents with no knowledge on nutrition (Table 4).

## Discussion

The aim of the study was to assess the prevalence of minimum dietary diversity and associated factors among lactating women in three districts of Haryana state. The consumption of a diverse range of foods has been linked to improved nutritional outcomes among women and children [27–29]. Dietary diversity is an important component in determining whether or not a diet is adequate and appropriate [30]. In the present study, approximately 77% of lactating women have adequate minimum dietary diversity, as a result are more likely to have sufficient or higher micronutrient consumption than their counterparts with inadequate MDD. To ensure optimal micronutrient sufficiency, the FAO recommended that all pregnant and lactating women achieve the minimum dietary diversity score [24]. To the best of knowledge, this is the first study from the selected study site. In another Indian study, 61.5% of the Indian women in a study by Soumya et al. on MDD were below the average dietary diversity score of 4.3 [31]. A similar type of studies were conducted in neighboring countries of Nepal [26] and Pakistan [30] and found 46.5 and 20% dietary diversity among lactating women, respectively, which are much lower than our estimates. However, another study from Haryana indicates similar results where dietary diversity amongst boys and urban adolescents was found to be high [32]. Moreover, a link has been established between income generation and dietary diversity in Haryana especially in farming households. Since the state is predominantly an agricultural state, this could be a reason for better dietary diversity than other studies [33]. Besides, our findings are consistent with findings by Lander et al. on Indian pregnant women, who found that 70% of them had appropriate dietary diversification [34].

In addition, with further classification into specific food groups, the typical diet of these women consists of cereals, pulses, other vegetables and fruits, and milk and milk products, and the consumption of animal based protein was very low (18%). This could be attributable to the culturally predominant vegetarian nature of the population [35]. According to a 2018 survey released by the Registrar General of India, Haryana is one of the top five states with the highest percentage of vegetarians [36]. Moreover, while controlling for potential confounding factors in multivariable analysis, the results of this study revealed that higher age, high educational level, and high nutritional knowledge of women remained significant predictors of minimum dietary diversity of respondents.

Lactating mothers with higher age groups showed significantly higher MDD compared with the lower age groups. A similar finding was reported by Gitagia and Colleagues [37] in Kenya. The explanation for these disparities could be that women with a higher age group are more aware of food options available locally and as they have grown they tend to sidestep the advice given by the neighbor or the elderly during the lactation period. Moreover, women get more autonomy in domestic decision-making as they get older [38] and hence can decide on their eating habits. According to Malhotra and Passi, the diets of young women were monotonous and cereal-based. Milk and milk products, legumes, green leafy vegetables, other vegetables, and fruits were all consumed in insufficient amounts [39]. However, in a study conducted in Sri Lanka [40], no association was identified between dietary score and age category, while in another study conducted in Ethiopia younger women had higher MDD than older women [41].

MDD was found to be associated with education level where lactating women with higher education had more diverse food. Lactating mothers who had education until graduation and above had a diet that was more than twice as diverse as those who had no schooling. This finding is comparable to that of prior studies conducted by Amugsi [42] with data from The Ghana Demographic and Health Survey and Nguyen in Bangladesh, Vietnam, and Ethiopia [16]. A possible explanation for the same could be that educated individuals have higher exposure and it is comparatively easier for them to receive appropriate information about varieties of food and understand the importance of increasing dietary diversity and meal frequency during lactation than uneducated mothers [43]. Education has also been shown to empower women to make independent decisions and have more access to household resources that are vital for their nutritional status [22, 23]. Less education, on the other hand, may be connected to poor food choices and preparation due to a lack of information and resources in conjunction with their dietary diversity [44].

Knowledge of nutrition has an impact on an individual’s diet [45]. Nutritional knowledge influences healthy food habits and ensures that nutrient requirement are met throughout the life cycle. Knowing your nutritional requirements makes it easier to make food choices that will improve your health and well-being by preventing nutrient excess or deficiency [46]. In this study, good nutritional knowledge predicted the minimum dietary diversity score of lactating mothers. Lactating women with no knowledge of nutrition when compared to their peers with strong nutrition knowledge, were twice likely to have inadequate dietary diversity. This result is comparable to the study conducted by FAO [47] and Getacher and colleagues [11]. This can easily be explained that women with more nutrition education understand the importance of a wide variety of foods and are more likely to consume these foods. Increased understanding among lactating women is also a result of appropriate antenatal and postnatal health care [11]. Increased knowledge about variety of food groups led the women to have adequate intake and consumption.

The limitations of the study cannot be overlooked. Dietary diversity was determined based on answers from participants’ recalls, which depended on memory and recall accuracy. The data is cross-sectional in nature hence causal inference cannot be established.

## Conclusions

The study found that the majority of lactating women in Haryana had attained minimum dietary diversity, which was linked to their socio-demographic characteristics and knowledge of nutrition. The study findings clearly demonstrated the importance of education, age, and nutritional knowledge in achieving the minimum dietary diversity and, as a result, enhanced nutrient intake among lactating women. In light of these findings, existing programmes like Integrated Child Development Services (ICDS), nutritional awareness platforms like VHNDs (Village Health and Nutrition Day) and AWC (Anganwadi Centers) must be supported and strengthened to improve the awareness of the population on nutritional related aspects. Such approaches would help to improve dietary diversity, especially among lactating women who have increased nutrients needs.

## Data Availability

The datasets analyzed in this study are available from the corresponding author on reasonable request.
